# Effects of prolonged photoperiod on growth performance, serum lipids and meat quality of Jinjiang cattle in winter

**DOI:** 10.5713/ab.20.0750

**Published:** 2021-02-15

**Authors:** Yan Yu, Jingyun Qiu, Jincheng Cao, Yingying Guo, Hui Bai, Shengjuan Wei, Peishi Yan

**Affiliations:** 1 Department of Animal Science and Technology, Nanjing Agricultural University, Nanjing 210095, China

**Keywords:** Gene Expression, Jinjiang Cattle, Meat Quality, Photoperiod, Serum Lipids

## Abstract

**Objective:**

This study was conducted to investigate the potential effects of prolonged photoperiod on the serum lipids, carcass traits, and meat quality of Jinjiang cattle during winter.

**Methods:**

Thirty-four Jinjiang bulls aged between 14 and 16 months were randomly assigned to two groups that were alternatively subjected to either natural daylight +4 h supplemental light (long photoperiod, LP) or natural daylight (natural photoperiod, NP) for 96 days. The potential effects on the levels of serum lipids, carcass traits, meat quality, and genes regulating lipid metabolism in the intramuscular fat (IMF) of the cattle were evaluated.

**Results:**

Jinjiang cattle kept under LP showed significant increase in both dry matter intake and backfat thickness. the serum glucose and the plasma leptin levels were significantly reduced, while that of melatonin and insulin were observed to be increased. The crude fat contents of biceps femoris muscle and longissimus dorsi muscle were higher in LP than in NP group. In longissimus dorsi muscle, the proportions of C17:0 and C18:0 were significantly higher but that of the C16:1 was found to be significantly lower in LP group. The relative mRNA expressions in IMF of longissimus dorsi muscle, the lipid synthesis genes (proliferator-activated receptor gamma, fatty acid-binding protein) and the fatty acid synthesis genes (acetyl-coa carboxylase, fatty acid synthetase, 1-acylglycerol-3-phosphate acyltransferase) were significantly up-regulated in LP group (p<0.05); whereas the hormone-sensitive lipase and stearoyl-CoA desaturase 1 were significantly down-regulated in LP than in NP group.

**Conclusion:**

Prolonged photoperiod significantly altered the growth performance, hormonal levels, gene expression and fat deposition in Jinjiang cattle. It suggested that the LP improved the fat deposition by regulating the levels of different hormones and genes related to lipid metabolism, thereby improving the fattening of Jinjiang cattle during winter.

## INTRODUCTION

Jinjiang cattle, a breed indigenous to China with zebu lineages, and revered for their great tolerance to roughage, have been reported to display excellent resistance to heat stress and provide a desirable meat quality [1,2]. However, Jinjiang cattle are relatively smaller in size and grow relatively slowly during winter [3].

In recent years, studies on fat deposition in Jinjiang cattle mainly focused on nutritional regulation [4], However, photoperiod management has been extensively tried to improve growth or production performance in domestic animals. Pigs in a long photoperiod (LP) group showed a greater live weight and carcass weight as compared to a short photoperiod (SP) group [5]. Moreover, the breasts from the boilers subjected to 23 L:1 D and 16 L:8 D had the greatest content of fat [6]. These results suggested that exposure to a prolonged photoperiod could influence lipid metabolism and production performance in different animals.

In ruminants, photoperiod management can be used to improve heifer growth and maximize accretion of lean tissue, including mammary parenchyma [7]. Steers in nature photoperiod (NP) produced fatter carcasses than those on LP, and heifers on NP deposited more fatty tissue between autumn and winter and less between winter and spring compared with those on LP [8]. In addition, LPs during established lactation increased milk production in dairy cattle and dairy sheep [9–13]. However Some researchers have found that compared with NP, short-day photoperiod during lactation significantly increased daily milk yield in goats and cows [14], Moreover, many scholars pointed out that the photoperiod manipulation on milk yield and milk composition were associated with mammary development and hormone changes [15,16].

Although several previous studies have extensively described the responses of physiological functions, energy metabolism and production performance of different mammals exposed to photoperiod manipulation, there is only limited information available on the photoperiod responses of beef cattle. Therefore, the effect of prolonged photoperiod on growth performance of Jinjiang cattle is worth exploring. In this study, the carcass characteristics, meat quality and gene expression under different photoperiods in Jinjiang bulls was evaluated. This study provides a certain scientific basis for photoperiod management of Jinjiang cattle in winter.

## MATERIALS AND METHODS

### Ethics statement

The care and use of animals followed Animal Research Institute Committee guidelines of Nanjing Agriculture University, China. This study has been approved by the Committee of the Animal Research Institute of Nanjing Agriculture University, China (SYXK2011-0036).

### Animals and light time

The experiment was carried out during November 8, 2017 to February 12, 2018 in Gao ‘a national beef cattle system high safety test station located in Jiangxi Province (lat. 24° 29′ to 30° 04′ N and 113° 34′ to 118° 28′E) and maintained at a range of −3°C to 17°C. A total of 34 Jinjiang bulls, aged 14 to 16 months-with body weights of 276.93±6.83 kg, were randomly divided into two groups. The control group continuously received the NP (12 h light from 0500 to 1700 and 12 h darkness); the treatment group received LP (16 h light from 0500 to 2100 and 8 h darkness). The two groups of cattle were housed in two semi-enclosed sheds and were fed and watered *ad libitum* under the normal conditions. The animals were acclimated to the experimental condition for 7 days prior to the start of the experiments. The whole experimental period lasted for a total of 96 days. The composition and nutrient levels of the basal diet is provided in Table 1.

### Blood sampling and analysis

The blood was collected once a week before the end of the experiment and at 4 h intervals. All the plasma samples were run in duplicate in a single assay. The plasma levels of glucose (Glu), total cholesterol (TC), low-density triglycerides (TG), non-esterified fatty acids (NEFA), high-density lipoproteins, low-density lipoprotein, and apolipoproteins (A1, B, E) were individually measured at the Clinical Biochemistry Service of Nan Jing general hospital (Nanjing, China), using Beckman AU5811 analyzer (Beckman-Coulter, 250S.Kraemer Boulevard Brea, USA). The plasma melatonin level was determined using high performance liquid chromatography (HPLC, Thermo Ultimate 3000; Thermo Fisher Scientific, Waltham, MA, USA) as described previously by Yin et al [16]. Leptin and insulin levels were measured in plasma using commercial bovine ELISA kit (Shanghai Enzyme-linked Biotechnology, Shanghai, China) according to the manufacturer’s instructions. For plasma insulin analysis, the results with intra-assay coefficient of variation (CV) of 3.0% to 6.0% were taken into consideration. For leptin analysis, results were acceptable with a CV of less than 5%.

### Meat sample collection and quality determination

At the end of the experiment, six cattle were randomly selected from each group for slaughter. The animals were weighed and transported to a meat processing plant where they were kept in individual boxes with access to water for 12 h. The backfat thickness and eye muscle area was determined by ultrasonic (Liaoning Hande B ultrasound HD-9300A; Dandong, China). The animals were stunned before slaughter. The carcasses were dressed and cut along the spine into two half-carcasses that were chilled for 45 min at 4°C. The meat quality and nutrient content of Jinjiang cattle was determined based on the method previously described by Zheng [17]. The samples from longissimus dorsi muscle (LDM) and biceps femoris muscle (BFM) were collected and stored at −20°C until further analysis. All slaughter and post slaughter processes were carried out in accordance with the current meat industry regulations.

### Fatty acid composition

One hundred milligram intramuscular fat (IMF) from LDM were prepared and extracted in chloroform: methanol (2:1, vol/vol), and non-adecanoic acid (N5252, 646-30-0; Sigma-Aldrich, St. Louis, MO, USA) was added as an internal standard. After methylation (NaOH/MeOH followed by HCl/MeOH), the fatty acids and thirty-seven standard fatty acid methyl esters (C13-CRM47885; Sigma-Aldrich, USA) were analyzed on gas chromatograph (Agilent 7890A, New York, USA) with a CP-Sil 88 column (100 m×0.25 mm×0.2 mm). The split ratio was 1:10. The oven temperature was maintained at 140°C for 2 min, and increased to 180°C at a rate of 1.5°C/min and maintained at 180°C for 10 min, and then increased to 240°C by 4°C and thereafter maintained at 240°C for 20 min.

### Relative mRNA expression analysis

The total RNA was extracted from IMF using Trizol Reagent as per the manufacturer’s instructions. The purity and concentration of total RNA were measured using a spectrophotometer (Nanodrop 2000; Thermo Fisher, USA) at 260 and 280 nm. The ratios of absorption (260/280 nm) of all samples were observed to be between 1.8 and 2.0. RNA was further treated with RNase-free DNase l to carefully remove contaminating genomic DNA. Total RNA was reverse transcribed to cDNA (10 μL reaction system was applied for 500 ng of total RNA) using a Prime Script RT Master Mix kit. Real-time polymerase chain reaction (PCR) was carried out in an optical 96-well plates on an ABI 7500 Real-Time PCR System (Applied Biosystems, Foster City, CA, USA) using SYBR Premix Ex Taq kits. The reagents used in this process were obtained from Takara Biotechnology Co. Ltd., Dalian, China. The list of primers used for real-time PCR assay have been presented in Table 3 and the primers were synthesized by BGI (Beijing Genomics Institution, Beijing, China). All the primers used in this study are listed in Table 2. The 2^−ΔΔCt^ method was used to analyze the relative changes in the gene expression after normalization against 18S ribosomal RNA used as an internal control.

### Statistical analysis

The data has been expressed as the mean±standard error of the mean. Statistical analyses was performed using SPSS Statistics 22 (SPSS, Inc., Chicago, IL, USA). Student’s t-test was generally employed for single sample sets statistical comparisons. The level of statistical significance was set bilaterally at 5%.

## RESULTS

### Growth performance

The possible effects of light on the growth performance of Jinjiang cattle are shown in Table 3. Interestingly, the cattle held under photoperiods of 16 h of light/d showed significantly increased dry matter intake (DMI) compared to the cattle held under NP (p<0.05); however, no significant differences were observed of average daily gain (ADG) and F/G (DMI/ADG) in both the LP and NP groups.

### Blood lipid metabolism related indicators

The effects of prolonged photoperiod on the blood lipid metabolism of Jinjiang cattle are shown in Table 4. We found that the Glu and TC levels in the treatment group were significantly reduced (p<0.001) but TG and other indexes were not significantly affected (p>0.05). The ApoE levels in the experimental group were significantly increased (p<0.001).

### Plasma hormone levels

**Figure 1:**
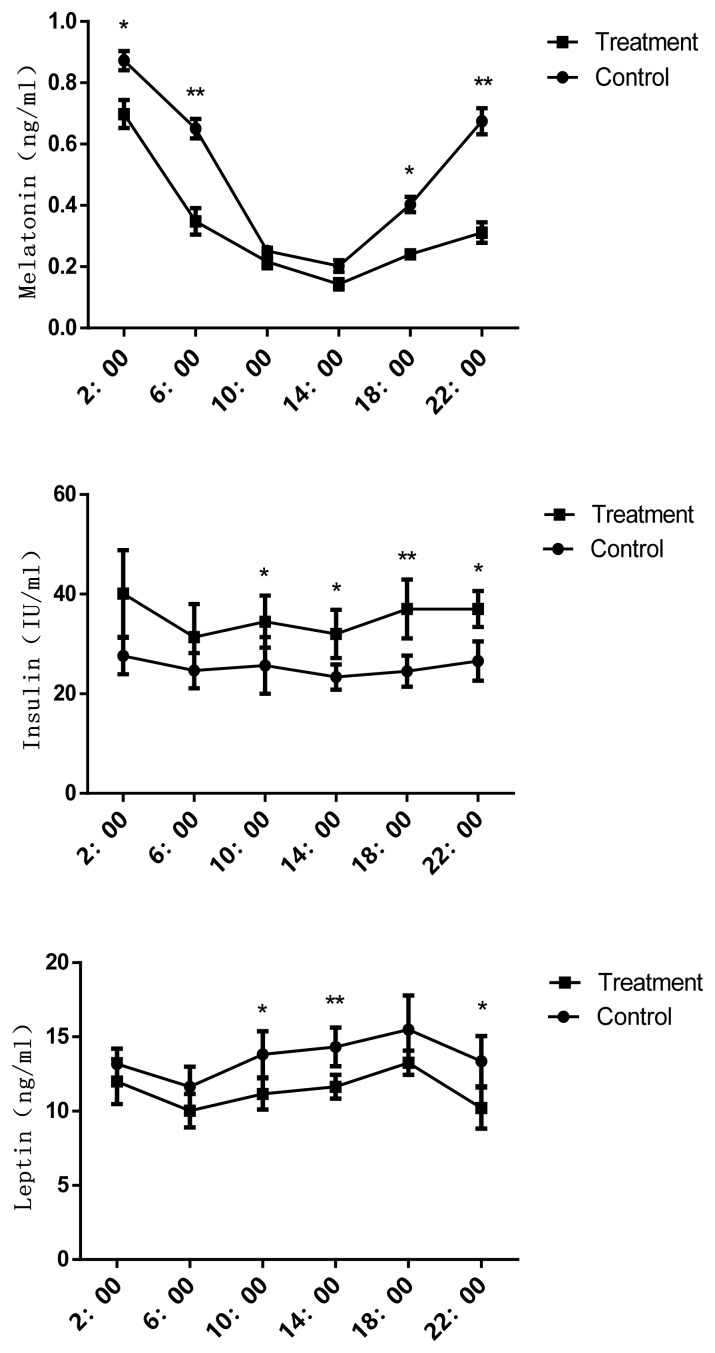
Effects of prolonged photoperiod on blood lipid metabolism related hormone levels in Jinjiang cattle in winter. The data has been shown as the mean±standard error of six replicates, * and ** mean values within different letters were significantly different (p<0.05) and (p<0.01).

The effects of prolonged photoperiod treatment in winter on the blood lipid metabolism related hormone levels in Jinjiang cattle are shown in Figure 1. Interestingly, melatonin levels were found to be lower during the daytime but higher at night in control and the treatment groups; melatonin levels in LP group were noted to be significantly lower than the control group at 6:00 and 22:00 (p<0.05). Moreover, it was observed that melatonin level decline was significantly higher in LP than those in NP group at 6:00. The levels of leptin were also significantly reduced (p<0.05) and insulin levels were found to be higher in LP group than in NP group (p< 0.05).

### Carcass traits and meat quality

The possible effects of the prolonged photoperiod carcass traits and meat quality of Jinjiang bulls are respectively shown in Table 5 and 6. The backfat thickness was found to be significantly higher in LP (p<0.001). There were no significant differences in BFM in meat quality at pH_45 min_, cooking loss, shear force and meat color (L* and b*) between the LP and NP groups (p>0.05). However, LDM in LP group displayed a significantly lower redness (p<0.05). In addition, the dripping loss of BFM increased significantly when cattle were exposed in LP (p<0.05). The possible effects of prolonged photoperiod on conventional nutrients content of Jinjiang cattle are shown in Table 7. It was found that a relatively longer photoperiod significantly increased the crude fat of the BFM in LP (p<0.05), but there was no significant difference observed both in the dry matter content and crude protein content in BFM and LDM (p>0.05).

### Fatty acid profile

The effects of prolonged photoperiod on fatty acid composition are shown in Table 8. The fatty acid composition of IMF in LDM from a prolonged light time up to 16 h was measured. It was found that the levels of C17:0 and C18:0 were significantly increased (p<0.05), while the levels of C16:1 were significantly decreased (p<0.05). However, C18:1 level was not significantly affected between the LP and NP groups (p>0.05), the proportions of saturated fatty acids, monounsaturated fatty acids (MUFA), and polyunsaturated fatty acids (PUFA) showed no significant differences between the LP and NP groups (p>0.05).

### Relative mRNA expression

**Figure 2:**
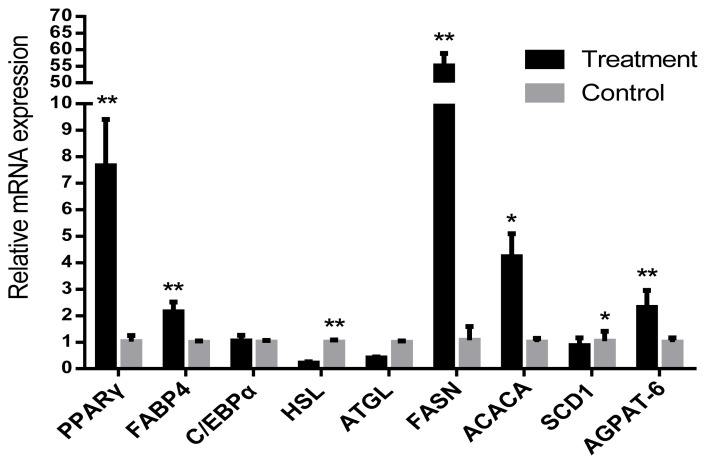
Relative mRNA expressions of lipid metabolism-related genes were normalized to 18s gene expression. The data has been shown as the mean±standard error of six replicates, * and ** mean values within different letters were significantly different (p<0.05) and (p<0.01).

The relative mRNA expression levels of lipid synthesis related genes proliferator-activated receptor gamma (*PPARγ*) and fatty acid-binding protein (*FABP4*) in IMF of LDM were significantly higher (p<0.05) than those in NP. However, the level of hormone-sensitive lipase (*HSL*) gene was significantly decreased (p<0.05). The fatty acid synthesis gene acetyl-coa carboxylase (*ACACA*), fatty acid synthetase (*FASN*), and 1-acylglycerol-3-phosphate O-acyltransferase (*AGPAT-6*) genes were also significantly increased in LP (p<0.05). The relative mRNA expression of stearoyl-CoA desaturase 1 (*SCD1*) were significantly decreased (p<0.05). CCAAT/enhancer binding protein (*C/EBPα*) and adipose triglyceride lipase (*ATGL*) were found to be not significantly different between the LP and NP groups (p>0.05) (Figure 2).

## DISCUSSION

### The potential effects of photoperiod on the growth performance and blood lipid metabolism related indicators

The length of photoperiod usually plays an important role in the growth, food intake and metabolism rhythm of animals [11,18,19,20]. Sheep exposed to a change from LP to SP reduce voluntary food intake [21]. However, extending the photoperiod for cattle in winter reduced body fatness in both steers and heifers and increased the time heifers spend lying down but that there were no major effects on growth rate or food intake [9]. In our experiments, it was found that the cattle held under LP showed significantly increased DMI relative to NP. However no significant differences were observed in ADG and F/G. This result was not consistent with previous study, which might due to different animal species or their physiological stage.

These results indicates that a longer photoperiod may affect the appetite levels and energy metabolism of Jinjiang cattle.

A number of previous studies have shown that *Phodopus sungorusx* appetite and body adiposity can substantially increase with associated central leptin insensitivity during a LP [22]. Leptin, which is mainly expressed and secreted by adipocytes, functions in the control of body mass through exerting its potential effects on both energy intake and energy expenditure [23]. Serum leptin concentration has been reported to dramatically decrease during winter-like acclimation, accompanied by an increase in energy intake in Brandt’s voles [24]. Furthermore, leptin receptors Ob-Ra and Ob-Rb mRNA were higher under long DL in lactating and pregnant Holstein cows [25]. As a result, the increased DMI may be due to hormonal regulation, in addition, it may also be caused by increased fat deposition that requires more energy.

Different hormones (such as insulin, leptin and melatonin) have been found to be closely associated with regulating lipid metabolism, and their content in the body may be directly or indirectly affected by the photoperiod [23]. In addition, both leptin and insulin levels may not only be directly affected by photoperiod, but can be also regulated by dietary energy levels and melatonin [26]. The activation of melatonin receptor 1 signaling at night can modulate insulin sensitivity during the day via the regulation of the phosphatidylinositol 3-kinase transcription and activity in mice [27]. Moreover, in Fischer 344 rats exposed to a SP of 6 h light/d showed an increased level of Glu and NEFA. The downstream post-receptor target of insulin was significantly down-regulated in 6 h light/d as compared to the 12 h light/d [28]. The lower Glu and the higher insulin level indicate that insulin can improve Glu utilization and enhance anabolism while significantly reducing the mobilization of body fat decomposition in the cold winter. Additionally, bulls’ backfat thickness was significantly increased the prolonged photoperiod treatment, which might be beneficial to withstand the low temperature for Jinjiang cattle. Moreover, pigs exposed to the longer photoperiod showed a greater tendency towards a higher slaughtering body weight and a higher saturation degree of subcutaneous fat in the raw thighs [29]. This suggests that fat deposition could also increase in Jinjiang cattle during the prolonged photoperiod treatment. Insulin can promote fat synthesis, whereas leptin may exert opposite effects. Hormonal changes noticed were generally consistent with the fat deposition, and these observations suggested that exposure to the longer photoperiod may regulate hormone levels thereby promoting fat deposition.

### Effect of photoperiod on meat quality of Jinjiang beef in winter

The BFM predominantly can control the activities of cattle, which are mainly affected by the amount of activity. Our previous study reported that the activity of Jinjiang cattle decreased with the rest time increased in LP group. The LDM can be used in high-end steaks, and the meat quality may be primarily affected by the dietary energy. Therefore, the effect of photoperiod on meat quality of these two different parts may be worth exploring in future studies. The longer photoperiod substantially increased the dipping loss in the BFM of Jinjiang cattle, but it had no significant effect on the LDM.

At 45 min after slaughter, the a* value of the LP group was found to be significantly lower in LDM of Jinjiang cattle, however no significant changes were noted in the meat color of BFM. Previous studies in broiler chickens reported that muscles from 20 L:4 D appeared to be lighter and more discolored, coupled with higher lipid oxidation and protein denaturation as compared to 12 L:12 D [30,31]. The broilers subjected to 23 L:1 D and 16 L:8 D had maximum fat and least protein [7]. It has also been reported that the contents of fat and protein of the loin from castrated crossbred ideal male lambs exposed to the LP were significantly affected. The water-holding capacity of the loin was found to be lowest when the animals were exposed to a LP, while no significant differences were observed for water-biding capacity and cooking losses [32]. Moreover, pigs in the LP group displayed a greater live weight and carcass weight as compared to the SP group; hams obtained from the LP group were significantly heavier and their weight losses during the dry-curing period were noted to be significantly reduced when compared to the SP group [6]. All the above results suggest that changes in meat quality may be significantly related to the muscle location and the species of the animal.

Although the muscle crude fat was increased, the meat quality was not significant changed. It may be due to the short feeding cycle and the amount of fat deposited is not at a level that significantly changes the meat quality. In addition, the measurement sample size is small, and the error is large. What is exciting to us is that the LP increases the muscle fat content, which is still significant in beef cattle production, but the effect on meat quality may need to be further verified by more experiments.

### Effect of photoperiod on fatty acid composition and the expression of genes related to lipid metabolism

The main MUFAs found in beef include C16:1 and C18:1, and main PUFAs are C18:2, C18:3, C20:4 [33]. Among the MUFAs, C18:1 has been primarily found to be a useful indicator for meat quality because of its association with beef palatability [34]. When Jinjiang cattle were raised in the LP, the levels of C17:0 and C18:0 were found to be significantly increased, while the levels of C16:1 were significantly decreased. Although the photoperiod had no significant effect on levels of C16:0 and C18:1, the level of C18:1 was higher in LP group. These findings indicated that Photoperiod could improve the fatty acid composition of Jinjiang cattle to some extent, and LP improved the flavor quality of Jinjiang beef substantially.

The SCD exists in adipose tissue and has been reported to be involved in cellular metabolism and the differentiation of precursor adipocytes. The amount of IMF content and the proportion of MUFA can increase spectacularly in grain-fed cattle with time on feed in relation to the activity of *SCD* [35]. The increased levels of C18:0 might be associated with the down-regulated *SCD1* in LP group. *ACACA* and *FASN* are the key regulatory enzymes involved in the lipid synthesis process. The relative mRNA expressions of *FASN* and *ACACA* genes were up-regulated in LP, and promoted the synthesis of fatty acids. In addition, therefore, photoperiod may mediate the expression of related genes to regulate fatty acid composition.

Changes in photoperiod length are transduced into neuroendocrine signals by melatonin secreted by the pineal gland triggering seasonally adaptive responses in many animal species. Siberian hamsters, transferred from a long-day ‘summer-like’ photoperiod to a short-day ‘winter-like’ photoperiod, exhibit a naturally occurring reversal in obesity [36]. Insulin, leptin and melatonin have all been reported to regulate lipid metabolism by controlling the expression of related genes, and some research showed that melatonin inhibits fat synthesis by reducing the mRNA expression level of key genes such as *PPARγ*, *C/EBPα*, and *C/EBPβ* of fat synthesis in mouse 3T3-L1 preadipocytes [37].

The relative mRNA expressions of lipid synthesis genes of *PPARγ*, *FABP4*, and *AGPAT* has been associated with TG synthesis which were similarly up regulated upon exposure to LP. The lipid hydrolysis gene HSL was significantly down-regulated in LP. This finding also suggested the possibility of the increased fat synthesis and reduced lipolysis at the molecular level in the cold environment.

There are two possible reasons for LP promoting fat deposition in Jinjiang cattle. On the one hand, LP may increase DMI and directly promote the expression of genes related to fat deposition. On the other hand, LP can directly or indirectly regulate the expression of genes related to lipid metabolism through hormones.

In this study, the effects of LP on the characteristics of lipid metabolism and related mechanisms of growth performance of Jinjiang cattle were preliminarily investigated, but the specific mechanisms are worth further investigation.

## CONCLUSION

Prolonged photoperiod had no significant effect on the growth performance and meat quality of Jinjiang cattle, however it showed a certain improvement trend. The LP significantly increased the muscle fat deposition level, which is of great significance in beef cattle production. This study provided a certain scientific basis for photoperiod management of Jinjiang cattle in winter.

## Figures and Tables

**Table 1:** Composition and nutrient levels of diet (DM basis)

Ingredient	Percentage	Nutrients	Content
	
Soybean meal	3.08	NE (MJ/kg)^2)^	2.74
Brewers grains	75.13	DM (%)	45.81
Corn	13.67	CP (%)	8.57
Straw	5.55	EE (%)	5.26
Premix^1)^	1.93	NDF (%)	47.33
Sodium bicarbonate	0.39	ADF (%)	23.92
Salt	0.25		
Total	100		

DM, dry matter; NE, net energy; CP, crude protein; EE, ether extract; NDF, neutral detergent fiber; ADF, acid detergent fiber.

1) Ingredients of premix: vitamin A 250,000 U, vitamin D_3_ 40,000 U, vitamin E 1,000 U, Cu (CuSO_4_5H_2_O) 1 g/kg, Fe (FeSO_4_7H_2_O) 5 g/kg, Mn (MnSO_4_ H_2_O) 4 g/kg, Zn (ZnSO_4_7H_2_O) 3 g/kg, Se (Na_2_SeO_3_) 0.01 g/kg, I (KI) 0.05 g/kg, Co (CoSO_4_7H_2_O) 0.01 g/kg, and Mg (MgSO_4_) 50 g/kg.

2) Calculated value.

**Table 2:** Sequences of primers used in quantitative polymerase chain reaction

Gene	GenBank ID	Forward primer sequence (5′ to 3′)	Reverse primer sequence (5′ to 3′)	Product size (bp)
*PPAR γ*	NM_181024.2	TGGAGACCGCCCAGGTTTGC	AGCTGGGAGGACTCGGGGTG	111
*FABP4*	NM_174314.2	TCCTTCAAATTGGGCCAGGAA	CCCTTGGCTTATGCTCTCTCA	218
*C/EBPα*	NM_176784.2	TGGGCAAGAGCCGGGACAAG	ACCAGGGAGCTCTCGGGCAG	166
*HSL*	NM_001080220.1	ATTGCCGACTTCCTACGAGA	AGTCCGATGGAGATGGTCTG	119
*ATGL*	FJ897536.1	TCTGCCTGCTGATTGCTATG	GGCCTGGATAAGCTCCTCTT	121
*FASN*	NM _001012669	GGTGCGTCCTGGTGTCTAA	CCTCGGGTGAGGACATTTAT	85
*ACACA*	AJ132890	CATCTTGTCCGAAACGTCGAT	CCCTTCGAACATACACCTCCA	101
*SCD1*	AY241933	TCCTGTTGTTGTGCTTCATCC	GGCATAACGGAATAAGGTGGC	101
*AGPAT6*	DY208485	AAGCAAGTTGCCCATCCTCA	AAACTGTGGCTCCAATTTCGA	101
*18S*	NR036642	GCCCGAAGCGTTTACTTTGA	TTCCATTATTCCTAGCTGCGGTAT	82

*PPAR γ*, proliferator-activated receptor gamma; *FABP4*, fatty acid-binding protein 4; *C/EBPα*, CCAAT/enhancer binding protein α; *HSL*, hormone-sensitive lipase; *ATGL*, adipose triglyceride lipase; *FASN*, fatty acid synthetase; *ACACA*, acetyl-coa carboxylase; *SCD1*, stearoyl-CoA desaturase 1; *AGPAT6*, 1-acylglycerol-3-phosphate O-acyltransferase.

**Table 3:** Effects of photoperiod on growth performance of Jinjiang cattle

Items	Control	Treatment	p-value
Average daily gains (ADG, kg/d)	0.369±0.036	0.456±0.063	0.245
Dry matter intake (DMI, kg/d)	5.49±0.19^b^	6.34±0.20^a^	0.007
F/G (DMI/ADG)	14.89±0.6	13.90±0.8	0.337

Data has been expressed as the mean±standard error of the mean (n = 9).

Means within a row with common superscripts do not differ (p>0.05).

Student’s t-test were performed to compare the values between the groups and significant differences were represented with different letters (^a,b^).

**Table 4:** Effects of photoperiod on serum lipid metabolism index of Jinjiang cattle

Items	Control	Treatment	p-value
Glucose (ng/L)	5.100±0.127^a^	4.450±0.067^b^	0.044
Total cholesterol (mmol/L)	5.463±0.150^a^	4.377±0.048^b^	<0.001
Triglycerides (mmol/L)	0.290±0.025	0.293±0.016	0.993
Non-esterified fatty acids (mmol/L)	0.180±0.006	0.190±0.006	0.976
High-density lipoprotein (mmol/L)	2.630±0.158	2.423±0.034	0.518
Low-density lipoprotein (mmol/L)	0.397±0.014	0.367±0.013	0.926
Apolipoprotein A1 (g/L)	0.060±0.003	0.043±0.002	0.958
Apolipoprotein B (g/L)	0.025±0.001	0.021±0.002	0.990
Apolipoprotein E (mg/L)	11.33±0.211^b^	16.67±0.211^a^	<0.001

Data are expressed as the mean±standard error of the mean (n = 6).

Means within a row with common superscripts do not differ (p>0.05). Student’s t-test were performed to compare the values between the groups and significant differences were represented with different letters (^a,b^).

**Table 5:** Effects of prolonged photoperiod carcass traits of Jinjiang cattle

Traits	Control	Treatment	p-value
Marbling score	2.07±0.201	2.50±0.224	0.183
Eye muscle area (cm^2^)	95.28±3.118	86.74±2.993	0.066
Backfat thickness (cm)	1.96±0.392^b^	3.06±0.460^a^	<0.001

Data are expressed as the mean±standard error of the mean (n = 6).

a,b Means within a row with common superscripts do not differ (p>0.05).

Student’s t-test were performed to compare the values between the groups and significant differences were represented with different letters.

**Table 6:** Effects of prolonged photoperiod on fleshy characteristics of Jinjiang cattle

Traits		Position	Control	Treatment	p-value
pH_45 min_		Biceps femoris muscle	5.516±0.101	5.442±0.081	0.569
		Dorsi longus muscle	5.419±0.112	5.281±0.133	0.433
Shear force (/kgf)		Biceps femoris muscle	3.907±0.236	3.944±0.157	0.897
		Dorsi longus muscle	3.594±0.108	3.775±0.127	0.288
Dripping loss (%)		Biceps femoris muscle	3.035±0.265^b^	4.498±0.364^a^	0.004
		Dorsi longus muscle	3.636±0.224	4.292±0.224	0.051
Cooking loss (%)		Biceps femoris muscle	29.7±1.810	33.72±1.63	0.109
		Dorsi longus muscle	28.4±2.010	29.4±1.621	0.694
Meat color	L*	Biceps femoris muscle	39.78±0.597	40.03±0.569	0.768
	a*		18.96±0.128	18.71±0.122	0.188
	b*		8.700±0.127	8.533±0.131	0.381
	L*	Dorsi longus muscle	39.70±0.416	39.87±0.470	0.796
	a*		18.93±0.101^a^	18.38±0.188^b^	0.028
	b*		8.867±0.102	8.367±0.201	0.051

Data are expressed as the mean±standard error of the mean (n = 6).

L*, lightness; a*, redness; b*, yellowness.

Means within a row with common superscripts do not differ (p>0.05).

Student’s t-test were performed to compare the values between the groups and significant differences were represented with different letters (^a,b^).

**Table 7:** Effects of prolonged photoperiod on conventional nutrients content of Jinjiang cattle

Items	Position	Control	Treatment	p-value
Dry matter (%)	Biceps femoris muscle	24.29±0.208	24.61±0.421	0.510
	Dorsi longus muscle	26.55±0.673	25.52±0.307	0.182
Crude fat % DM	Biceps femoris muscle	4.25±0.294^b^	5.36±0.355^a^	0.037
	Dorsi longus muscle	6.72±0.257	7.78±0.403	0.051
Crude protein % DM	Biceps femoris muscle	23.47±0.202	23.52±0.109	0.832
	Dorsi longus muscle	23.41±0.310	22.71±0.255	0.112

DM, dry matter.

Data are expressed as the mean±standard error of the mean (n = 6).

a,b Means within a row with common superscripts do not differ (p>0.05).

Student’s t-test were performed to compare the values between the groups and significant differences were represented with different letters.

**Table 8:** Fatty acid composition (% of total fatty acids) in intramuscular fat of Jinjiang cattle

Items	Control	Treatment	p-value
C10:0 Capric acid	0.04±0.012	0.046±0.010	0.709
C12:0 Lauric acid	0.051±0.008	0.053±0.005	0.788
C13:0 Tridecanoic acid	0.020±0.006	0.013±0.004	0.384
C14:0 Myristic acid	1.870±0.262	1.530±0.201	0.327
C15:0 Pentadecanoic acid	0.341±0.029	0.310±0.037	0.538
C16:0 Palmitic acid	25.722±1.124	25.270±1.251	0.794
C17:0 Heptadecanoic acid	1.660±0.081^b^	1.880±0.051^a^	0.044
C18:0 Stearic acid	17.200±0.782^b^	19.740±0.793^a^	0.047
C20:0 Arachidic acid	0.261±0.017	0.402±0.161	0.407
C14:1 Myristoleic acid	0.174±0.089	0.231±0.100	0.663
C15:1 Pentadecenoic acid	0.151±0.060	0.280±0.082	0.229
C16:1 Palmitoleic acid	1.480±0.121^a^	1.060±0.120^b^	0.033
C17:1 Heptadecenoic acid	0.260±0.089	0.320±0.061	0.590
C18:1 Oleic acid	41.210±2.110	41.699±2.090	0.872
C20:1n9 Eicosenoic acid	0.141±0.078	0.162±0.609	0.975
C22:1n9 Erucic acid	0.069±0.004	0.088±0.008	0.064
C18:2n Linoleic acid	6.890±0.430	5.100±0.681	0.051
C18:3n3 γ-Linolenic acid	1.320±0.152	0.880±0.249	0.971
C18:3n6 α-Linolenic acid	0.491±0.209	0.420±0.301	0.852
C20:3 Eicosatrienoic acid	0.041±0.023	0.041±0.009	0.968
C20:4 Arachidonic acid	0.025±0.002	0.020±0.002	0.054
C20:5 Eicosapentaenoic acid	0.181±0.029	0.187±0.018	0.842
C22:6 Docosahexaenoic acid	0.012±0.003	0.015±0.003	0.482
SFA	47.252±5.125	49.316±5.629	0.274
MUFA	43.489±4.231	43.832±2.022	0.176
PUFA	8.959±2.152	6.662±1.651	0.417

SFA, saturated fatty acids; MUFA, monounsaturated fatty acids; PUFA, polyunsaturated fatty acids.

Data are expressed as the mean±standard error of the mean (n = 6).

Means within a row with common superscripts do not differ (p>0.05).

Student’s t-test were performed to compare the values between the groups and significant differences were represented with different letters (^a,b^).
